# Downregulation of THRSP Promotes Hepatocellular Carcinoma Progression by Triggering ZEB1 Transcription in an ERK-dependent Manner

**DOI:** 10.7150/jca.51657

**Published:** 2021-05-19

**Authors:** Qiong Hu, Xiaolu Ma, Chuner Li, Chenhao Zhou, Jiayao Chen, Xuechun Gu

**Affiliations:** 1 Department of Laboratory Medicine, Zhoushan Hospital of Zhejiang Province, Zhoushan, Zhejiang, 316021, China.; 2 Department of Clinical Laboratory, Fudan University Shanghai Cancer Center; Department of Oncology, Shanghai Medical School, Fudan University, Shanghai, 200032, China.

**Keywords:** Hepatocellular carcinoma, Thyroid hormone responsive gene, epithelial to mesenchymal phenotypes, HCC therapies

## Abstract

**Background:** Hepatocellular carcinoma (HCC) is a major leading cause of cancer mortality worldwide. Thyroid hormone responsive (THRSP) gene is primarily known for regulating responses to thyroid hormones, but its expression has been correlated with differential outcomes in some cancers. To date, however, its role in the progression of HCC remains unknown.

**Methods:** The mRNA and protein expression of THRSP was measured in HCC tissues and cell lines via qPCR and western blot assays. Lentiviral transfection was used to establish stable cell lines overexpressing THRSP and shRNA was used to silence THRSP. The effects of THRSP on cell growth were then determined *in vivo* and *in vitro*. Cell migration and invasion of HCC cells were investigated using transwell and wound healing assays.

**Results:** In tissue samples from patients, HCC tissues had decreased THRSP expression relative to adjacent healthy tissues. Further, patients with decreased THRSP protein and mRNA expression had worse outcomes. Knockdown of THRSP led to increased cell growth, migration, and invasion of HCC cells, and THRSP overexpression exerted an anti-tumor effect *in vivo* and *in vitro*. We found that increased expression of THRSP inhibited hepatocellular carcinogenesis by inhibiting the process of epithelial-to-mesenchymal transition through acting on the ERK/ZEB1 signaling pathway.

**Conclusion:** THRSP may act as a functional tumor suppressor and was frequently reduced in HCC tissue samples. We identified a novel pathway for the THRSP/ERK/ZEB1-regulated suppression of HCC tumorigenesis and invasion. Restoring THRSP expression may represent a promising approach for HCC therapies.

## Introduction

Hepatocellular carcinoma (HCC), the most common type of primary liver cancer, ranks as the fifth leading cause of cancer mortality worldwide 1. Surgical resection remains the preferred curative therapy for HCC patients. Recently, the potential combination of immunotherapies and targeted therapies has provided additional treatment alternatives for HCC. However, the high frequency of tumor metastasis or recurrence still exceeds 50%, largely due to the significant heterogeneity observed in HCC 2-5. Therefore, it is essential to understand the biological mechanisms and related signaling pathways that are associated with HCC progression in order to improve the prognosis and survival of HCC patients.

The thyroid hormone responsive (*THRSP*), also known as spot 14, was originally identified as a regulator that responds to thyroid hormones 6. This gene was important for epithelial cell proliferation and intracellular lipogenesis in the mammary glands 7. Wellberg et al. previously reported that the *THRSP* exerted extensive amplification in breast cancers and was highly expressed in breast tumor tissues 8. Indeed, the high expression of THRSP in primary invasive breast cancer was proved to predict the time to tumor recurrence, with greater expression associated with less recurrence-free survival. Moreover, THRSP can also mediate progesterone-induced adipogenesis and promote the growth of breast cancer cells 9-11. Recently, studies have shown that THRSP is highly expressed in human liver tissues. It has been reported that THRSP, given its role in regulating lipid metabolism, plays an important role in the hepatocytes and is involved in the pathogenesis of nonalcoholic fatty liver disease 12-13. However, the role or mechanism of THRSP in the progression of HCC remains unclear.

In this study, data from public databases and biological experiments were combined to investigate the mechanism by which THRSP influences liver cancer. In contrast with prior findings in breast cancer, we observed that THRSP expression was frequently reduced in HCC and that down-regulation of THRSP was positively correlated with recurrence, larger tumor size, and more aggressive phenotype. Mechanistically, THRSP inhibited hepatocellular carcinoma progression by inhibiting the ERK/ZEB1 pathway induced EMT process.

## Materials and Methods

### HCC tissues

We recruited 10 pairs of HCC tissue samples, and corresponding adjacent non-cancerous tissue samples, along with related clinical information from patients undergoing surgery at the department of oncology, Fudan University Shanghai Cancer Center between December 2019 and February 2020. Signed informed consents were obtained from all subjects.

### Cell culture

L02, Huh7, HepG2 and Hep3B cells were purchased from the Institute of Biochemistry and Cell Biology (Chinese Academy of Sciences, Shanghai, China), and HCCLM3 cells were obtained from the Shanghai Zhongshan Hospital, Fudan university. All cell lines were confirmed to be free of mycoplasma contamination. The cells were maintained in Dulbecco's modifed Eagle's medium (DMEM, Gibco, Thermo Fisher Scientifc, Cambridge, MA, USA) supplemented with 10% bovine growth serum, 100 U/mL penicillin and 100 μg/mL streptomycin (Gibco) and cultured in an incubator at 37 °C, with 5% CO2.

### Lentivirus

Lentiviruses for THRSP overexpression were constructed by Merdobio Corporation (Shanghai, China). Those carrying the THRSP shRNA, were purchased from Santa Cruz Biotechnology (Santa Cruz, CA, USA). For stable cell line construction, Huh7 cells were transfected with THRSP-overexpressing or Hep3B cells with THRSP-silencing lentivirus at a multiplicity of infection (MOI) of 20, followed by selection using 2.5 µg/ml puromycin for 2 months. Non-stable clones were removed due to poor tolerance to puromycin.

### Western blot

Western blotting was performed following previous studies 14. The utilized antibodies included anti-THRSP (1:500, sc-516102; Santa Cruz), anti-phospho-ERK (Tyr202/Tyr204) (1:1000, 4377S; CST), anti-ERK (1:1000, 4695S; CST), anti-Slug (1:1000, 9585S; CST), anti-Snail (1:1000, 3879S; CST), anti-Twist (1:1000, 46702S; CST), anti-ZEB1 (1:1000, 70512S; CST), anti-ZEB2 (1:1000, 3396S; CST). anti-TGF-beta (1:1000, 5544S; CST), anti-AKT (1:1000, 4685S; CST), anti-phospho-AKT (1:1000, 4060S; CST), anti-E-cadherin (1:1000, 14472S; CST), and anti-N-cadherin (1:1000, 13116S; CST), anti-Vimentin (1:1000, 5741S; CST), Anti-GAPDH (1:3000, 5174S; CST) was used as a loading control.

### Real-time PCR

Total mRNA was extracted using the RNA Extraction Kit (Takara Bio Inc., Shiga, Japan) following the manufacturer's protocol. Reverse-transcription assays were performed using the PrimeScript™ RT Reagent Kit (Takara Bio Inc., Shiga, Japan). Then qRT-PCR was carried out using SYBR Premix Ex Taq™ (Takara Bio Inc., Shiga, Japan). All the primers were listed in Supplementary Table 1.

### Cell counting Kit-8 (CCK-8) assays

Stably infected cells were cultured in 96-well plates and maintained in incubator at 37 °C for overnight. Next day, the cell supernatant was removed and replaced with a fresh medium, containing the CCK-8 reagent (10 μL/well) and the complete DMEM medium (90 μL/well). After 2 h, the absorbance value was measured at 450 nm.

### Wound-healing assays

Stably infected cells were cultured in 6-well plates and maintained until cell fusion occurred. The bottom of the plate was scratched with a 100 μL pipette tip. The floating cells were washed out and pictures of wound closure were taken at 0 and 24 h, respectively.

### Colony formation assays

Stably infected cells were seeded in 6-well plates. After 14 days, the cells were fixed with 4% paraformaldehyde and stained with purple crystal. The size of the surviving colonies was observed and calculated. Photographs of colonies were collected.

### Transwell migration and invasion assays

The invasion and migration ability of cells were studied using Matrigel-coated (BD Biosciences, Franklin Lakes, NJ, USA) or non-Matrigel-coated Transwells. The serum was used as chemoattractant. Thus, Stable cell lines were cultured in the upper chamber of the transwells and supplemented with serum-free medium. DMEM medium supplemented with 10% FBS was added into the lower plate wells. After 24 hours of incubation, the upper filter with the cancer cells was removed and the cells that had migrated to the lower chamber were fixed, stained, and counted.

### Mouse model of HCC

Male BALB/c nude mice (4-6 weeks old) were purchased from the Experimental Animal Center of Shanghai Institute. Subcutaneous xenograft tumors were established as follows: 5 × 10^6^ cells were diluted in 0.15 mL of phosphate buffered saline and then injected subcutaneously into the flanks of nude mice. The size of the subcutaneous tumor was measured once per week as: length×width×width×0.5. After 6 weeks, the animals were sacrificed. The volume of the tumor was measured. Animal care and experimental protocols were conducted under guidelines approved by the Institutional Animal Care and Use Committee (IACUC) at Shanghai Cancer Center, Fudan University.

### Statistical analysis

All experiments were repeated at least three times in order to confirm the results. The data are reported as mean ± standard error of the mean (SEM). The Student's t-test or Wilcoxon matched-pairs test used SPSS 22.0 (SPSS, Chicago, IL, USA) software and Prism 6.0 (GraphPad Software, La Jolla, CA, USA). The difference was considered significant at P < 0.05.

## Results

### Low expression of THRSP predicts poor prognosis in HCC

**Figure 1:**
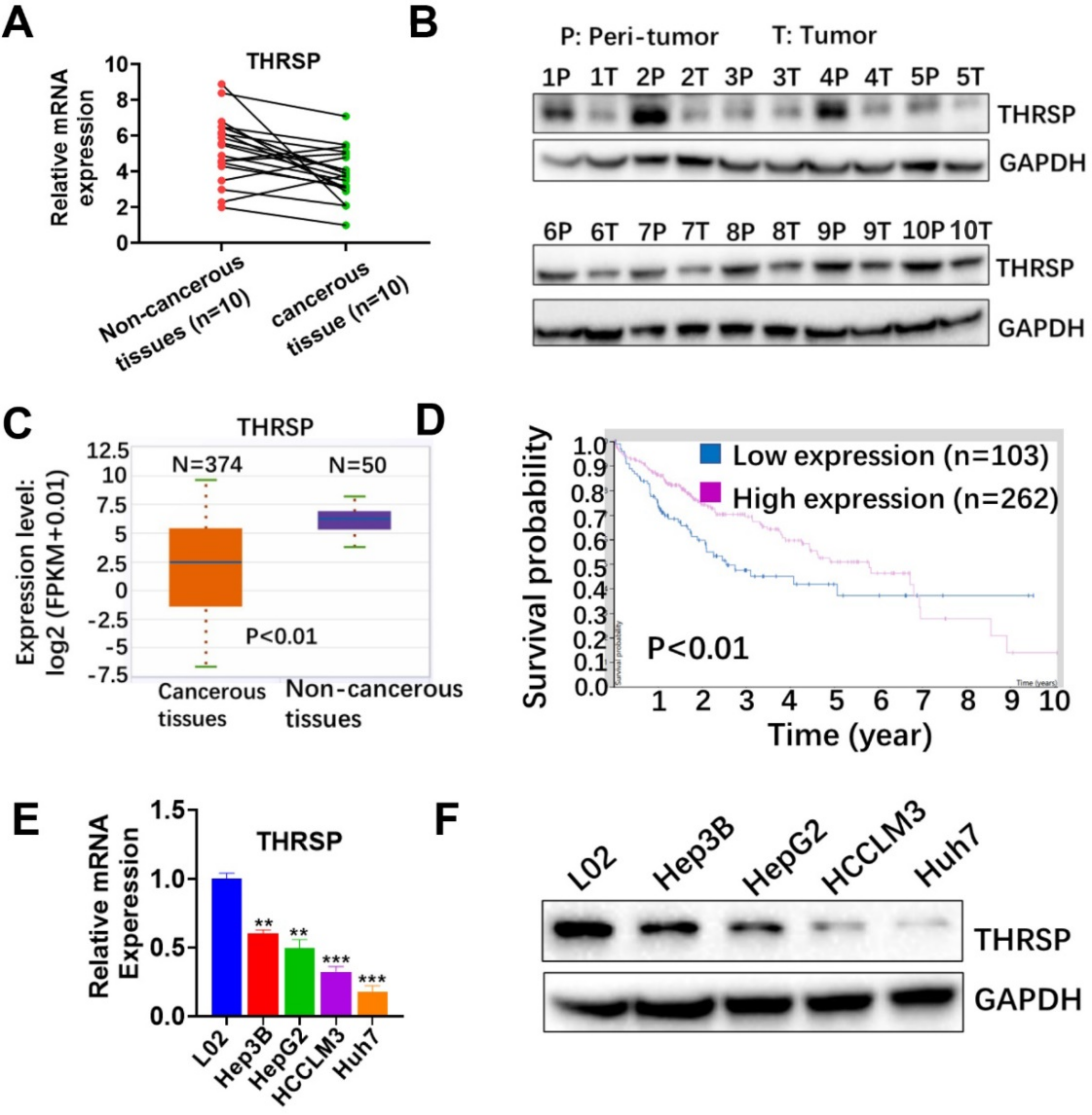
** THRSP is downregulated in human HCC and predicted poor prognosis.** A. mRNA levels of THRSP were analyzed in 10 HCC tissue samples, compared with adjacent non-cancerous samples. B. Protein levels of THRSP were analyzed in HCC tissue samples and adjacent non-cancerous tissues. C. Relative THRSP expression levels in 374 HCC patients and 50 normal samples from The Cancer Genome Atlas database. D. A Kaplan-Meier analysis of overall survival (OS) in patients with different expression level of THRSP. E. Relative THRSP levels in L02 and four HCC cells were analyzed using qRT-PCR assay. F. Relative THRSP levels in L02 and four HCC cells were analyzed using western blotting. Data are means ± SD of three independent experiments. *p < 0.05, **p < 0.01, ***p < 0.001.

To verify the clinical significance of THRSP in HCC, we first determined the expression of THRSP in both cancer tissues and paired adjacent non-cancerous tissues using qRT-PCR and western blot (WB). We found that mRNA and protein expression of THRSP were significantly decreased in the majority of cancerous tissues, compared with paired non-cancerous tissues (Fig. 1A and 1B). These results were consistent with data from the TCGA database (http://starbase.sysu.edu.cn/panCancer). According to TCGA database, mRNA expression of THRSP was down-regulated in 374 patients with liver cancer, compared with 50 normal samples (P<0.01, Fig 1C). Additionally, HCC patients with low THRSP expression had worse overall survival than patients with high THRSP expression according to TCGA database (Protein Atlas database (https://www.proteinatlas.org; Fig. 1D). Moreover, downregulation of THRSP was validated by qPCR and WB analyses in liver cancer cell lines relative to normal liver cells (Fig. 1E and 1F). These data indicated that THRSP was frequently decreased in HCC and low THRSP levels were related with poor prognoses.

### THRSP suppresses cell proliferation in HCC

**Figure 2:**
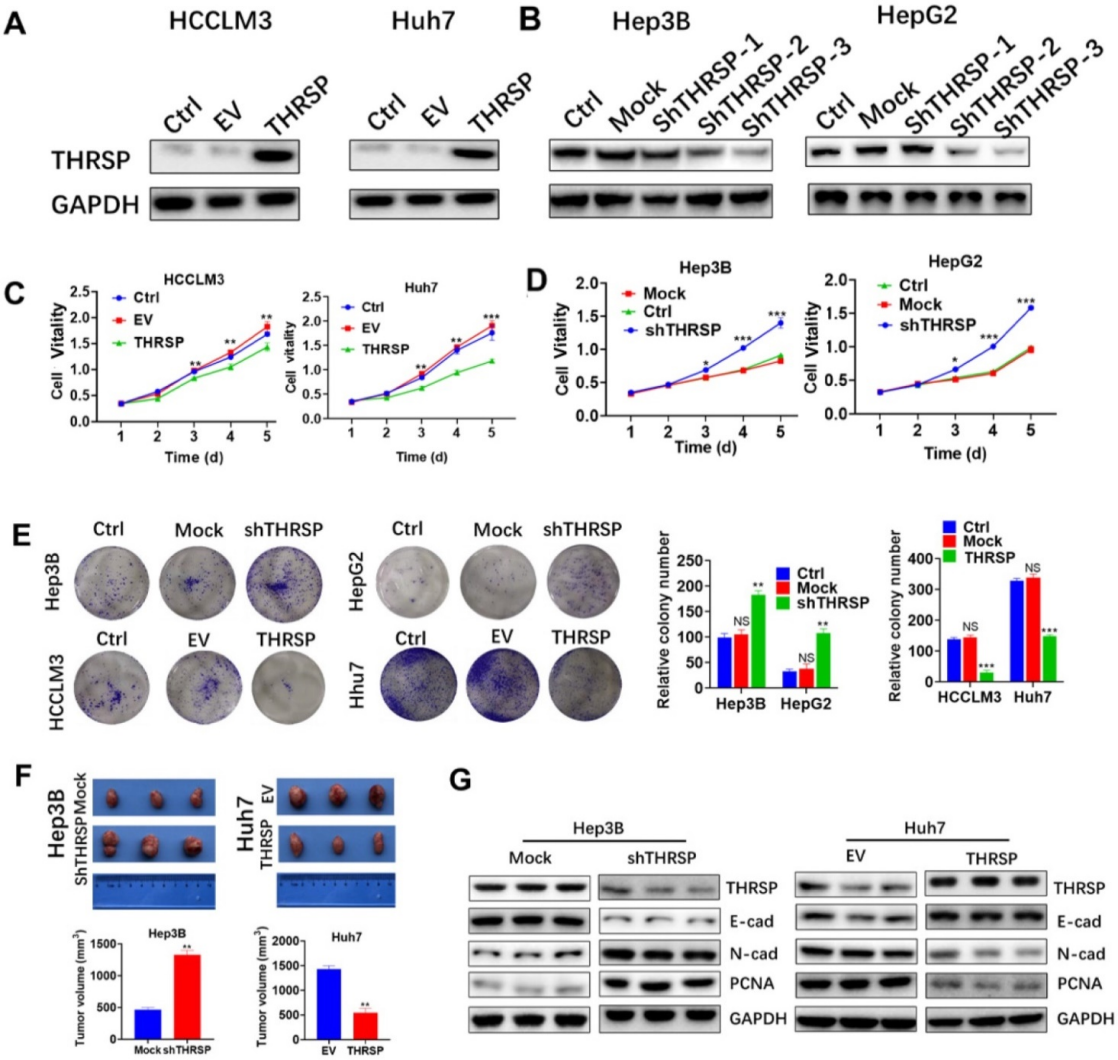
** THRSP inhibits HCC cell proliferation and tumorigenesis *in vitro* and *in vivo*.** A, B. Western blot analysis of the transfection efficiency of THRSP in HCC cell lines. C. Proliferation rate was analyzed using CCK-8 assay of HCC cells with THRSP knockdown. D. Proliferation rate was analyzed using CCK-8 assay of HCC cells with THRSP overexpression. E. Proliferation rate was analyzed by clone formation assay of HCC cells with THRSP overexpression or silenced. F. Downregulation of THRSP decreased Hep3B cell subcutaneous growth, whereas THRSP overexpression had the opposite effect. G. The EMT-related markers and proliferation-related markers were analyzed in the indicated tissues using WB assays. Data are means ± SD of three independent experiments. *p < 0.05, **p < 0.01, ***p < 0.001.

To further determine the biological effect of THRSP in HCC cells, we transfected HCCLM3 and Huh7 cell lines (THRSP-low) with lentivirus to overexpress THRSP. In addition, stable models of THRSP knockdown in Hep3B and HepG2 cell lines (THRSP-high) were established with short hairpin RNAs (shRNA). The efficiency of transfection was determined by RT-PCR and WB assays. We found that THRSP was successfully overexpressed (Fig. 2A), and shTHRSP-3 led to significant silencing, as expected (Fig. 2B). Therefore, shTHRSP-3 was used for following experiments. Meanwhile, the normal cells were used as control groups (Ctrl). *In vitro* assays showed that the overexpression of THRSP restrained the activity of HCCLM3 and Huh7 cells and inhibited proliferation, as shown in a CCK8 experiment (Fig. 2C). In contrast, downregulation of THRSP promoted proliferation of Hep3B and HepG2 cells (Fig. 2D). Similar result was also observed in Huh7 cells. Colony formation analyses demonstrated that overexpression of THRSP can decrease the number of clones of HCCLM3 and Huh7 cell lines, however silencing THRSP expression increased the number of clones formed by Hep3B and HepG2 cell lines (Fig. 2E).

To further confirm the above findings *in vivo*, subcutaneous xenograft tumors were constructed in nude mice. Significantly larger volumes of HCC tumors were found in the Hep3B-shTHRSP group, whereas smaller tumors were observed in the Huh7-THRSP group, compared with their corresponding control group respectively (Fig. 2F). To further demonstrate that THRSP suppressed tumor cell growth, we measured molecular markers of cell proliferation and invasion in the subcutaneous xenograft tumors. We found THRSP knockdown decreased expression of E-cadherin, but increased expression of N-cadherin and PCNA. In contrast, THRSP overexpression increased expression of E-cadherin, and inhibited expression of N-cadherin and PCNA. (Fig. 2G). In summary, our results suggested that THRSP inhibits HCC growth both* in vitro and in vivo*.

### THRSP suppresses cell migration and invasion of HCC

**Figure 3:**
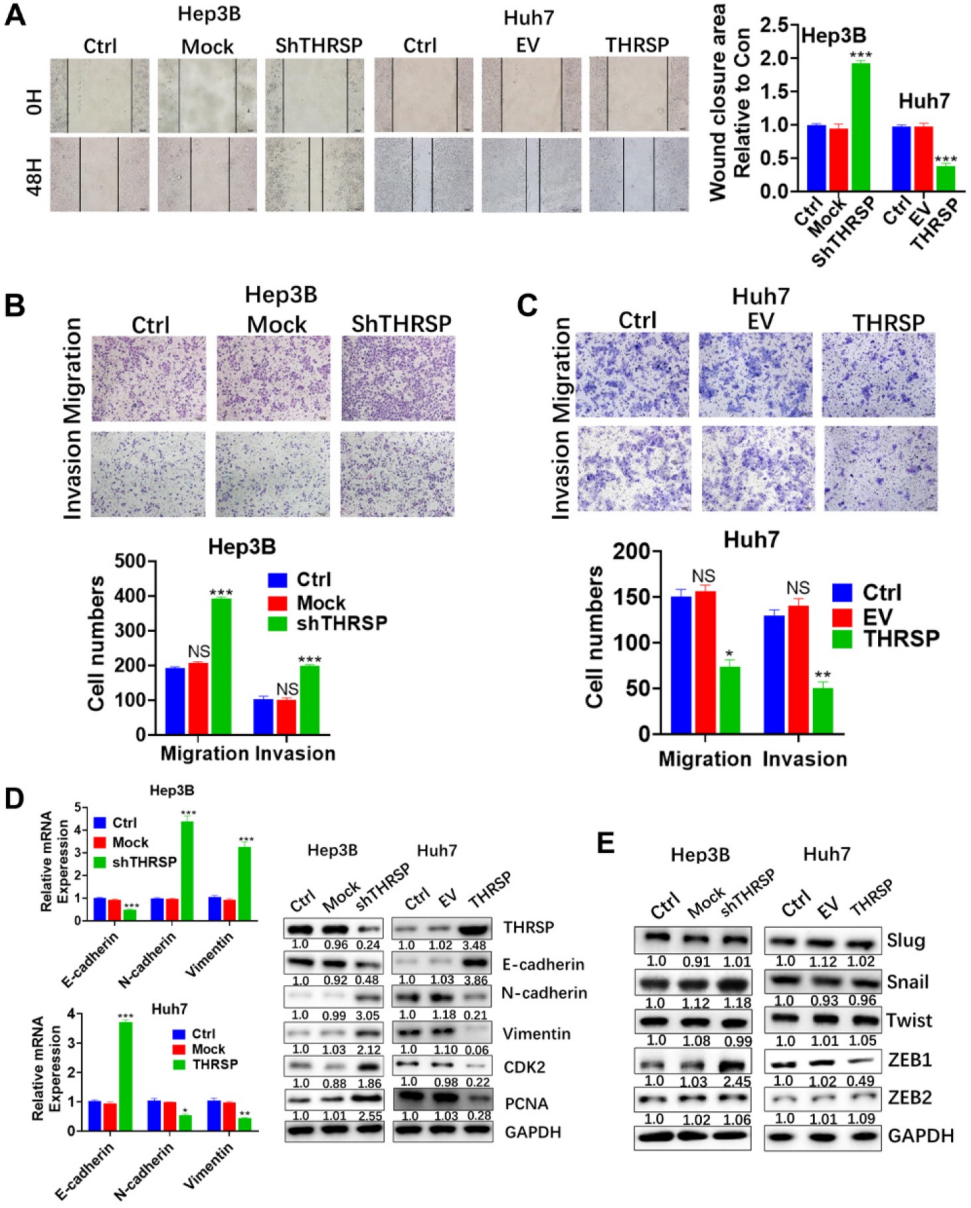
** THRSP inhibits HCC cell migration and invasion.** A. The migration of cancer cells was determined by the wound-healing assay. B, C. The migration and invasion ability were determined using transwell assays in the indicated cell lines. Scale bars: 100μm. D. mRNA levels of EMT-related markers were measured by qPCR. E. Protein levels of EMT-related markers and proliferation-related markers were measured by western blot. Data are means ± SD of three independent experiments. *p < 0.05, **p < 0.01, ***p < 0.001.

The impact of THRSP on the invasiveness and the mobility of HCC cells was explored. Wound-healing experiments showed that downregulation of THRSP increased mobility (as indicated by regrowth into the scratched area) in Hep3B and Huh7 cells, but overexpression of THRSP inhibited scratch regrowth (Fig. 3A). Further, THRSP-knockdown cells showed increased invasiveness and the mobility in Transwell assays, and THRSP overexpression decreased the invasive and migratory behavior of HCC cells (Fig. 3B and 3C).

Epithelial to mesenchymal transformation (EMT) is responsible for the migration and invasion of individual cancer cells 15. To clarify the underlying role of THRSP in the process of EMT, qPCR and WB were carried out to determine the expression of EMT-related markers. When THRSP was downregulated in Hep3B cells, the expression of E-cadherin (an epithelial marker) was decreased and the expression of N-cadherin and vimentin (mesenchymal markers) were increased, as were cell proliferation-related markers, CDK2, and PCNA. Conversely, THRSP overexpression increased the expression of E-cadherin and reduced that of N-cadherin and vimentin in Huh7 cells, as well as CDK2 and PCNA, compared to control cells (Fig. 3D). Moreover, we analyzed expression of the upstream transcription regulators of EMT, such as snail, slug, ZEB1/2, and twist, which are important for cell migration and invasion 16-18. Of these, only ZEB1 expression was decreased or increased with overexpression or downregulation of THRSP, respectively (Fig. 3E).

### THRSP-induced HCC progression was dependent on ERK phosphorylation

**Figure 4:**
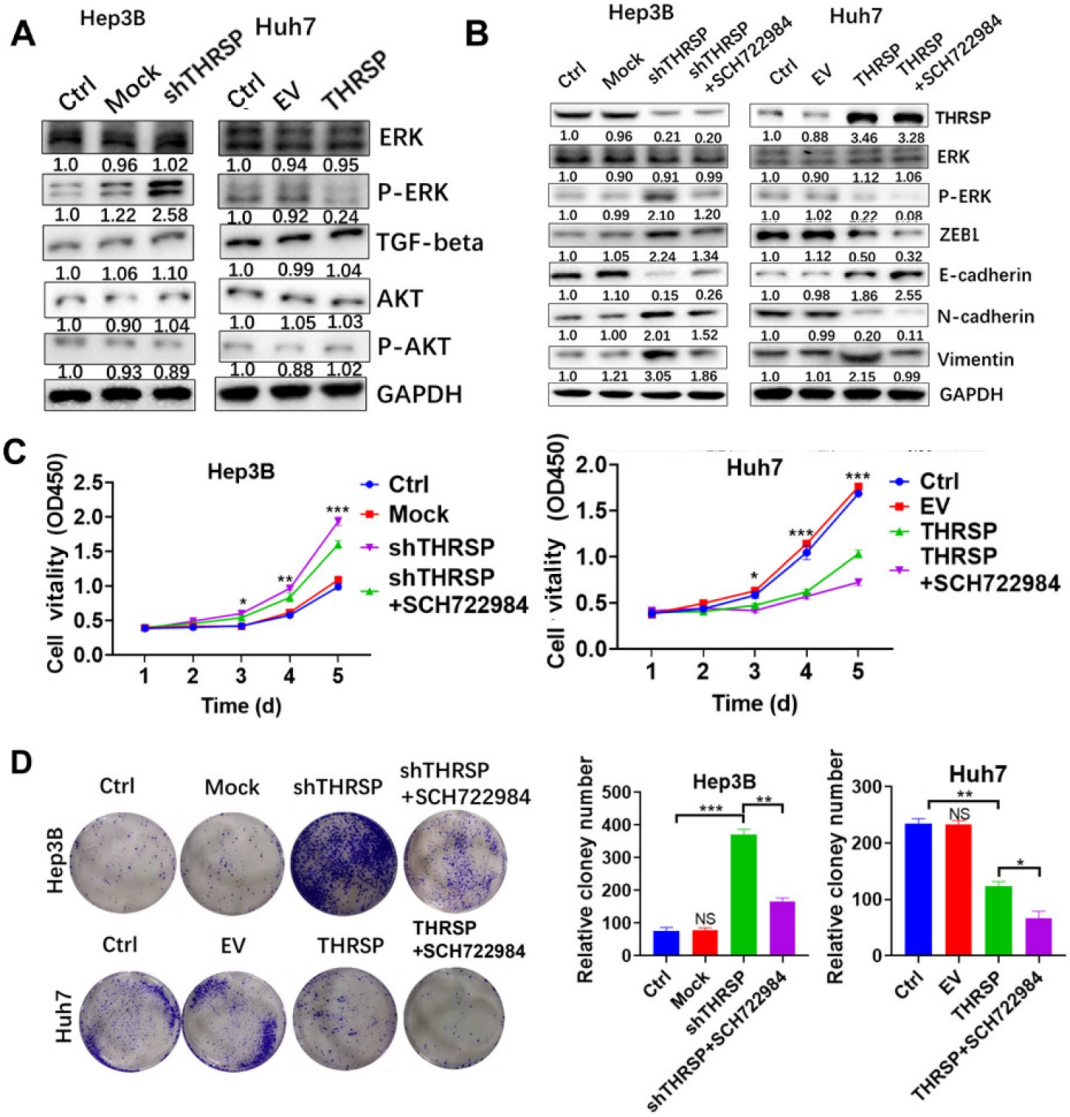
** THRSP reverses EMT process in an ERK/ZEB1 dependent manner.** A. Protein levels of signaling pathways were analyzed by western blot after modulation of THRSP expression. B. Western blot analysis of indicated markers in liver cancer cells. C. Proliferation rate was analyzed using CCK-8 assay in indicated HCC cells with SCH722984 treatment. D. Proliferation rate was analyzed using colony formation assay in indicated HCC cells with SCH722984 treatment. Data are means ± SD of three independent experiments. *p < 0.05, **p < 0.01, ***p < 0.001.

**Figure 5:**
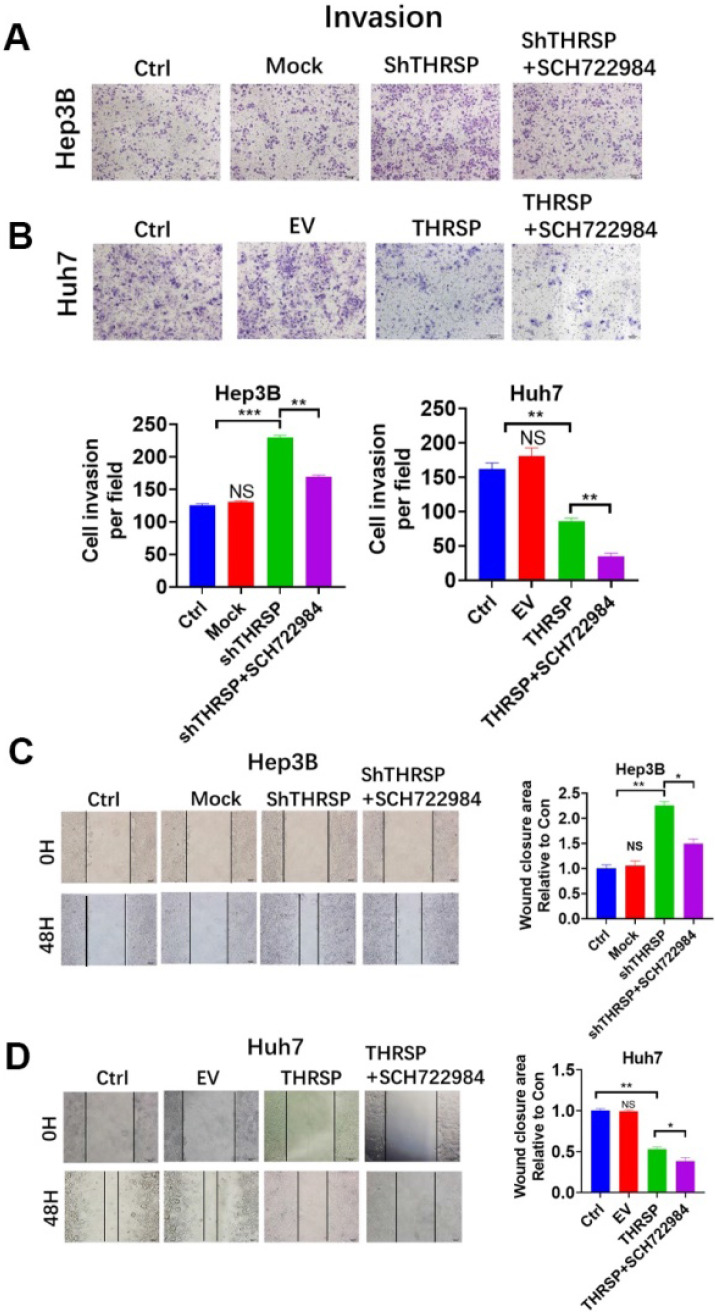
** THRSP abolished cell migration of HCC in an ERK/ZEB1 dependent manner.** A. Transwell analysis of Hep3B cell lines in THRSP knockdown cells with SCH722984 treatment. B. Transwell analysis of Huh7 cell lines in THRSP overexpression cells with SCH722984 treatment. C. Wound healing analysis of Hep3B cell lines in THRSP knockdown cells with SCH722984 treatment. D. Wound healing analysis of Huh7 cell lines in THRSP overexpression cells with SCH722984 treatment. Scale bars: 100 µm; Data are means ± SD of three independent experiments. *p < 0.05, **p < 0.01, ***p < 0.001.

Next, we sought to clarify the molecular mechanism by which THRSP regulates EMT. We found that the expression of phosphorylated ERK was increased after THRSP silencing and decreased after THRSP overexpression. However, there were no changes in p-AKT or TGF-β1 (Fig. 4A). We used SCH722984, a novel specific inhibitor of ERK1/2 19, 20, to explore the mechanisms of THRSP-induced HCC progression. Western blots demonstrated that the activation of p-ERK was inhibited after treating Hep3B-shTHRSP cells with SCH722984 (Fig. 4B). Consistently, the same effect was observed in Huh7-THRSP cells (Fig. 4B). CCK8 assays showed that the survival rate of cells was dramatically reduced after treatment with SCH722984 (Fig. 4C). Similarly, this was also confirmed in colony formation assays (Fig. 4D). When treated with SCH722984, the invasion and migration of Hep3B-shTHRSP and Huh7-THRSP cells were partially inhibited (Fig. 5A and 5B). Wound healing assays also confirmed that THRSP suppressed cell migration (Fig. 5C and 5D). These data indicate that the ERK signaling pathway is vital for THRSP-induced HCC progression and that ZEB1 is a key molecule in THRSP-induced EMT.

## Discussion

This study is the first to demonstrate a role of THRSP in the progression of HCC. Our evidence suggests that the expression of THRSP was significantly decreased in HCC tissues and HCC cell lines compared with adjacent non-cancerous tissues and normal liver cell lines. Patients with low levels of THRSP had shorter survival time than those with high levels of THRSP. Consistent with these clinical findings, we found that THRSP inhibited the proliferation, invasion, and migration of liver cancer cells* in vitro.* Further, *in vivo* assays confirmed that the growth of liver cancer cells was significantly suppressed.

EMT is a critical process in tumorigenesis, especially tumor migration and invasion 15. A variety of biological and pathological processes are attributed to EMT, such as tissue regeneration, embryo formation, and tumorigenesis 21, 22. Increasing amounts of evidence have demonstrated that EMT is responsible for the migration and invasion of different cancer types, including HCC 23, 24. However, there is a need for better understandings of the specific mechanisms of EMT progression. Additionally, targeted strategies for the clinical management of HCC in response to patient-specific EMT-related biomarkers remain an area for improvement 25. Previous studies have shown that P53 has an inhibitory effect on the EMT process 26. We found that downregulation of THRSP decreased epithelial markers and increased mesenchymal markers, thereby promoting EMT in HCC cell lines regardless of P53 mutation states. This indicated THRSP might inhibit the aggressive phenotype of HCC in an independent P53 manner. Importantly, among several transcription factors studied that is involved in the EMT process, only ZEB1 appeared to be affected by THRSP. This is the first report of the potential mechanism of THRSP in HCC.

Numerous previous studies have indicated that cancer signaling pathways are aberrantly activated to increase EMT and enhance carcinogenesis 27-29. We investigated several key pathways, including ERK1/2, TGF-β, and AKT signaling, and found that, when THRSP was silenced or overexpressed, only ERK signaling was activated, with altered protein levels of phosphorylated ERK but no change in total ERK protein expression. The ERK pathway is associated with the process of EMT 29, 30; the over-activation or inactivation of ERK signaling is frequently found in various cancers, including HCC 31-33. In our study, an underlying link between THRSP and the activation of ERK signaling in HCC cells was established. When THRSP-overexpressing cells were treated with an inhibitor of ERK1/2, the EMT process and tumorigenesis were partially inhibited. This suggests that the ERK pathway is important for THRSP-regulated HCC processes.

We found that THRSP reversed the EMT process in a manner that was dependent on the ERK/ZEB1 signaling pathway. Consistently, Sheng et al. have previously reported that the ERK/ZEB1 signaling pathway is essential for EMT 34. MAP kinase is known as an extracellular signal-regulated kinase (ERK) that acts as an integration point for a variety of biochemical signals and participates in multiple cellular processes such as proliferation, invasion, and transcriptional regulation. After activation, it moves into the nucleus to phosphorylate nuclear targets 32. Additionally, it can act as a transcriptional repressor, independent of its kinase activity 35. ZEB1 is a member of the zinc-finger E-box-binding homeobox family and is a transcription factor that regulates EMT. According to prior reports, ZEB1 promotes tumor invasion and metastasis by inducing EMT in many cancers. ZEB1 recruits a variety of chromatin enzymes from the E-cad promoter, which is a key mechanism for regulating EMT 36. Our *in vitro* assays showed that THRSP decreased ZEB1 expression but did not impact other transcription factors that also control EMT such as slug, snail, twist, or zeb2. ERK signaling was the only EMT-related pathway that appeared to be regulated by THRSP. Our experiments confirm that ERK modulates the expression of ZEB1. However, the specific regulatory relationship between ERK and ZEB1 remains a topic for further study.

In conclusion, we found that THRSP may act as tumor suppressor gene in HCC. Our results indicated that THRSP was decreased in HCC tissues and cell lines, and THRSP expression was negatively correlated with HCC progression and prognosis. THRSP inhibited cell proliferation, migration, and invasion in an ERK/ZEB1-dependent manner. This is the first report of the role of THRSP in HCC, and these findings suggest that restoration of THRSP expression might represent a promising therapeutic strategy towards HCC treatment.

## Supplementary Material

Supplementary table S1.

## Abbreviations

THRSP: Thyroid hormone responsive
ZEB1: zinc finger E-box binding homeobox 1
HCC: hepatocellular carcinoma
mRNA: messenger RNA
qPCR: Quantitative real-time polymerase chain reaction
MOI: multiplicity of infection
ERK: extracellular signal-regulated kinase
p-ERK: phosphorylated extracellular signal-regulated kinase
EMT: epithelial to mesenchymal transformation
ZEB2: Zinc finger E-box binding homeobox 2
TGF-beta: transforming growth factor-beta
MAP kinase: Mitogen-activated protein kinase
DMEM: Dulbecco's Modified Eagle's medium
CCK-8: Cell Counting Kit-8
FBS: fetal bovine serum
WB: western blot
shRNA: short hairpin RNA
PCNA: Proliferating cell nuclear antigen
CDK2: Cyclin-dependent kinase 2
