# Supplementary material for: Downregulation of THRSP Promotes Hepatocellular Carcinoma Progression by Triggering ZEB1 Transcription in an ERK-dependent Manner
Source: J Cancer. 2021 May 19;12(14):4247–56. doi: 10.7150/jca.51657 (PMC8176411; doi:10.7150/jca.51657)
Supplement: Supplementary file 1 — Supplementary table S1. [file jcav12p4247s1.pdf]

Supplementary Table 1. The primer sequences were used in study.

Gene		Sequences
THRSP	Forward	5′- CAGGTGCTAACCAAGCGTTAC -3′
	Reverse	5′- CAGAAGGCTGGGGATCATCA -3′
GAPDH	Forward	5′-GTCATCCAACGGGAATGCA-3′
	Reverse	5′-TGATCGGTTACCGTGATCAAAA-3′
E-cadherin	Forward	5’-CGAGAGCTACACGTTCACGG-3’
	Reverse	5’-GGGTGTCGAGGGAAAAATAGG-3’
N-cadherin	Forward	5’-AGCTCCATTCCGACTTAGACA-3’
	Reverse	5’-CAGCCTGAGCACGAAGAGTG-3’
Vimentin	Forward	5’-GCCCTAGACGAACTGGGTC-3’
	Reverse	5’-GGCTGCAACTGCCTAATGAG-3’
